# Liver toxicity during temozolomide chemotherapy caused by Chinese herbs

**DOI:** 10.1186/1472-6882-14-115

**Published:** 2014-03-30

**Authors:** Thomas Melchardt, Teresa Magnes, Lukas Weiss, Michael Grundbichler, Michael Strasser, Clemens Hufnagl, Martin Moik, Richard Greil, Alexander Egle

**Affiliations:** 1 Department of Internal Medicine III, Salzburg Cancer Research Institute, Paracelsus Medical University Salzburg, Austria, Müllner-Hauptstrasse 48, Salzburg 5020, Austria; 2 Department of Internal Medicine I, Paracelsus Medical University Salzburg, Austria, Müllner-Hauptstrasse 48, Salzburg 5020, Austria

**Keywords:** Temozolomide, Chinese herbs, Interaction, Liver toxicity, Hepatitis

## Abstract

**Background:**

Complementary and alternative medicine is often used by patients with malignant glioma. Although several interactions of various alternative agents with chemotherapy are known, none has been described for temozolomide so far.

**Case presentation:**

We report the case of severe liver toxicity with jaundice during radiochemotherapy with temozolomide likely due to interaction with a popular Chinese herbal formula after surgery for glioblastoma. After cessation of the herbal formula as well as the chemotherapy liver enzymes slowly normalized. Due to tumor progression the patient was retreated with temozolomide for 5 cycles without toxicity. Because of further progression combination treatment of bevacizumab and irinotecan was started and again no liver toxicity was observed.

**Conclusions:**

We conclude that the observed toxicity with jaundice was probably caused by an interaction of this popular Chinese formula and temozolomide. This is the first report about a relevant interaction of temozolomide and any herbal formula.

## Background

Complementary and alternative medicine (CAM) is frequently used by glioblastoma patients [1] but treating physicians are often left uninformed about its use. These patients are potentially at increased risk for herb-drug interactions resulting in increased toxicity due to higher serum levels of cytostatics on the one hand or undertreatment due to decreased efficacy caused by lower serum levels on the other hand.

## Case presentation

A 56 year old female Caucasian patient was diagnosed with a temporal-parietal glioblastoma WHO IV° after further evaluation for cephalea. Complete resection was performed using 5-aminolevulinic acid guided surgery and postoperative magnetic resonance investigation showed a small rest tumor. The patient had a Karnofsky performance status scale of 90% after resection without neurological deficiencies and was referred to our department for further treatment with radiotherapy and temozolomide (TEM). Co-medication consisted of dexamethason 4 mg per day because of edema, pantoprazol, levetiracetam and mirtazapine because of depression. Further medical history consisted of hypertension and recurrent cutaneous herpes infections. Adjuvant concomitant radiochemotherapy (RCT) was started with an absolute dose of 140 mg TEM per day 4 weeks after primary resection. Furthermore, prophylactic valaciclovir was prescribed. Treatment was initially well tolerated and the steroid was tampered. Unfortunately, TEM had to be stopped after 32 days of treatment and 24 fractions of radiotherapy because of grade II° thrombopenia and increasing liver enzymes. Mirtazapine, valaciclovir and pantoprazol were paused and radiotherapy was completed as planned after 30 fractions. Because of further increasing liver enzymes and jaundice (bilirubin: 16.7 mg/dl; AST: 263 U/l; ALT: 585 U/l) the patient was hospitalized and underwent further evaluation including exclusion of viral hepatitis, Epstein-Barr virus or cytomegalovirus-infection, computed tomography scan as well as autoantibody screening to exclude an autoimmune hepatitis. Endoscopic retrograde endoscopy excluded pathology of the biliary or pancreatic ductal system. A negative herpes polymerase chain reaction ruled out systemic herpes infection and levetiracetam was reduced in the absence of seizures with the intention to reduce co-medications.

**Figure 1:**
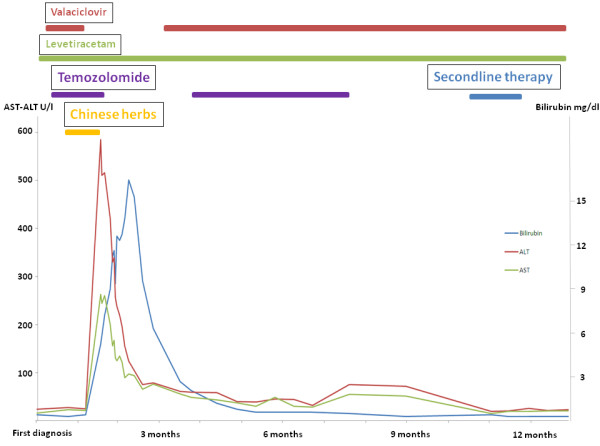
Course of liver enzymes during primary radiochemotherapy with temozolomide and re-exposition for 5 cycles after first diagnosis of glioblastoma.

After repeated and detailed conversations the patient reported the use of Chinese herbs as a decoction twice daily for 2 weeks between the beginning of the RCT and the diagnosis of increasing liver enzymes. Following the diagnosis of glioblastoma a general practitioner specialized in CAM recommended the use of a modified form of the Chinese herbal formula called Bu Zhong Yi Qi Wan (11 different herbs) compiled by a local pharmacy, which is commonly used in Chinese CAM. Four weeks after cessation of TEM and the Chinese herbs the liver enzymes started to decrease, but it took about 3 months to normalization of the bilirubin level (Figure 1).

Unfortunately, a [18 F]-fluorethylenthyrosin positron emission tomography positive residual tumor was detected 2 months after the end of radiotherapy. Therefore, despite normalization of the liver parameters was still pending we decided to restart TEM after repeated briefing of the patient to omit any CAM, because we were convinced that this toxic hepatitis was primarily caused by the addition of this Chinese herbal formula. Thus, TEM with an absolute dose of 250 mg per day for 5 days every 4 weeks was re-introduced, supportive medication with valaciclovir and 500 mg bi-daily levetiracetam remained unchanged. We continued treatment for 5 cycles of TEM without any increase of the liver parameters or any other toxicity except for mild cytopenia. Thereafter the patient did not want to continue treatment despite response, because she desired to undergo further complementary treatment. Unfortunately, 3 months after cessation of therapy symptomatic progression due to large edema was diagnosed and combination treatment of bevacizumab and irinotecan was initiated. No liver toxicity or any other severe side effect was observed after 3 months of treatment.

## Conclusion

Liver toxicity is a known but very rare side effect of TEM [2]. Reexpositions to TEM in our patient showed that toxic hepatitis was not causally related to TEM treatment alone or caused by the combination with valaciclovir or levetiracetam. Thus, we conclude that the observed liver toxicity was caused by the concomitant intake of the popular Chinese herbal formula, Bu Zhong Yi Qi Wan, in combination with TEM. Some direct hepatotoxicity by the herbal formula itself seems unlikely, because in traditional Chinese medicine Bu Zhong Yi Qi Wan is thought to be protective to the liver, but cannot be completely ruled out. To exclude an unknown toxic product in the formula we asked for some remnants of this formula, but unfortunately it was totally used up by the patient. Furthermore, the Chinese herbs were recommended by a physician not involved in the management of the patient and probable not familiar with GBM. Due to that we were primarily not aware of this possible interaction and this delayed clarification of this case.

This herbal formula consists of several different herbs and the exact composition varies frequently in CAM. The detailed list of herbs taken by our patient is shown in Table 1 and comprises at least two herbs Huang Qi and Huang Qin with relevant cytochrome P_450_ 3A4 (CYP3A4) inhibition [3,4]. The effect and safety of temozolomide was not addressed in patients with severe hepatic impairment in clinical trials, but no dose adjustment is recommended in these patients according to manufacturer’s labeling. TEM is nonenzymatically converted to the active alkylating metabolite in all tissues and excreted with the urine, therefore CYP isoenzymes are thought to play a minor role up to now. Nevertheless, the distribution of single nucleotide polymorphisms of the CYP450 system is different in Chinese compared to other populations [5]. This would in part explain, why such an interaction is observed in Europe but not in China so far despite integration of TEM in the national insurance schedule in China [6]. Furthermore, Chinese medicine comprises hundreds if not thousands of preparations. It may well slip the attention of physicians if a broad background of intake is assumed.

**Table 1:** Modified form of the Chinese herbal formula called Bu Zhong Yi Qi Wan

**Chinese name**	**Name and species**
Dang Shen	Radix Codonopsitis
Zhi Gan Cao	Radix Glycyrrhizae
Bai Zhun	Rhizoma Atractylodes macrocephala
Huang Qi	Radix Astragali
Chen Pi	Citrus reticulata
Chai Hu	Radix Bupleuri
Sheng Ma	Rhizoma Cimicifugae
Dang Gui	Radix Angelicae sinensis
Ge Gen	Radix Puerariae
Du Zhong	Cortex eucommiae
Huang Qin	Radix Scutellariae

Furthermore, liver toxicity due to pantoprazol or mirtazapine seems unlikely, because they were started 6 weeks before the start of RCTX without any toxicity and the overall low incidence of liver toxicity of these drugs.

Nevertheless, while the exact mechanism remains unclear, we still observed a severe liver toxicity with the combination of TEM and a popular Chinese formula, which is not reported up to now in the literature. In face of the frequent use of CAM and Chinese herbs in patients with malignant glioma we want to alert cancer physicians about this possible side effect.

We hope to inspire the discussion about the safety of any herbal formulas in combination with cytotoxic therapies and encourage physicians to seek faithful conversations dealing with the use of CAM in cancer patients. There are several reports on the detrimental effects of CAM on the efficacy of chemotherapeutic agents such as St. John’s wort with irinotecan or imatinib [7] or ascorbic acid and green tea with bortezomib [8,9] to name but a few. Therefore, we think that a comprehensive management of our patients should also include education about the risk of interactions with herbal formulas.

## Consent

We state that patient’s privacy is not compromised by this report and informed consent for publication was obtained.

## Competing interests

T.Me. and L.W. received travel support from Roche. R.G. and A.E. received speaking fees, research support and travel support from Roche. All other authors declare that they have no competing interests.

## Authors’ contributions

TMe, TMa, RG and AE are primarily responsible for writing the manuscript. All authors treated the patients, wrote and critically revised the manuscript.

## Pre-publication history

The pre-publication history for this paper can be accessed here:

http://www.biomedcentral.com/1472-6882/14/115/prepub
